# Ratiometric Impedance Sensing of Fingers for Robust Identity Authentication

**DOI:** 10.1038/s41598-019-49792-9

**Published:** 2019-09-19

**Authors:** Hyung Wook Noh, Chang-Geun Ahn, Hyoun-Joong Kong, Joo Yong Sim

**Affiliations:** 1 Medical Information Research Section, Welfare & Medical ICT Research Department, Electronics and Telecommunications Research Institute, Daejeon, 34129 Republic of Korea; 2 Department of Biomedical Engineering, Chungnam National University College of Medicine, 266 Munwha-ro, Jung-gu, Daejeon, 35015 Republic of Korea

**Keywords:** Biomedical engineering, Electrical and electronic engineering

## Abstract

We present a novel biometric authentication system enabled by ratiometric analysis of impedance of fingers. In comparison to the traditional biometrics that relies on acquired images of structural information of physiological characteristics, our biological impedance approach not only eliminates any practical means of making fake copies of the relevant physiological traits but also provides reliable features of biometrics using the ratiometric impedance of fingers. This study shows that the ratiometric features of the impedance of fingers in 10 different pairs using 5 electrodes at the fingertips can reduce the variation due to undesirable factors such as temperature and day-to-day physiological variations. By calculating the ratio of impedances, the difference between individual subjects was amplified and the spectral patterns were diversified. Overall, our ratiometric analysis of impedance improved the classification accuracy of 41 subjects and reduced the error rate of classification from 29.32% to 5.86% (by a factor of 5).

## Introduction

Biometric authentication relies on the individual unique biological characteristics (fingerprint, vein, iris, retina, face, etc.) of a person^1^. Biometric systems play an important role in personal, national, and global security by significantly improving personal identification and authentication^2^. Most current biometric technologies are based on the structural features of acquired images, which have many advantages such as convenience and simplicity^3^. However, the image-based biometrics methods also have their weaknesses^4^. For example, some image features, such as those of the fingerprint and iris, which are used in a wide variety of applications ranging from smartphones to immigration identity authentication, could be easily spoofed and its anti-fake performance is threatened^5^. For example, it is possible to spoof the fingerprint scanner by using a printed gelatin mold over a real finger because this technology can fail to discriminate an artificial fingerprint^6^. There are also potential threats for iris-based systems. Recently, the feasibility of some attacks have been reported, and it is actually possible to trick the iris recognition system with a printed iris, photo iris, and well-made color lens^7^. Overall, current biometrics technologies could not reach a balance between counterfeiting and usability, and therefore, its popularization remains limited^8^.

Under these challenges mentioned above, researchers have been seeking new alternatives to existing methods. Many novel and unconventional features, such as ear contour^9^, palm print^10,11^, nose pore^12,13^, vein patterns^14,15^, finger-knuckle-print^16,17^, and multimodal approaches have been adopted for development as new biometrics methods^18,19^. In recent years, new approaches such as biomedical engineering technologies have been proposed to provide non-image-based frequency or time domain information. For example, electroencephalography (EEG) and electrocardiogram (ECG) have been considered as new biological features in biometrics research. ECG-based technology has been reported as a biometric feature that provides strong liveness evidence^20,21^. EEG has also been studied for its potential for biometrics^22^, and Fingelkurts *et al*. has shown EEG oscillations that constituted EEG states were characteristic for 13 different groups of conditions in accordance to oscillations’ functional significance^23^. These studies are significant in that non-image-based biological features such as EEG and ECG can be adopted to develop new biometric methods, because of their robustness to attacks. However, these biological signals are still unsuitable for practical applications due to their limited accuracy and long recognition times as well as the fact that they are highly dependent on a relaxation or excitability of the subject compared to that required in traditional biometric technologies^24^.

In contrast, bioelectrical impedance spectroscopy and impedance tomography is a biomedical technique that measures the physiological state of living tissue and is less sensitive to emotional conditions^25^. It could also considerably vary from person to person owing to the intrinsic variability of the passive electrical properties of tissues and cells depending on the distribution of intracellular and extracellular fluid (ICF and ECF) as well as the movement of ions within tissues^26^. In *in vivo* human applications, the physiological state is typically measured using metal electrodes placed on the skin around an anatomic location of interest^27^. These electrical properties include information about the presence of specific tissue types (e.g., blood, muscle, bone, etc.), anatomical configuration (i.e., the direction and amount of skeleton and muscle), and tissue status^28^. There are significant impedance differences between various tissue types, anatomical configurations, and tissue states, each of which can provide a unique mechanism to distinguish people^29^. Besides, as the electrical response of body tissues relies on the frequency of the applied signal, impedance analysis can be performed over a broad frequency band^30^, which helps to better identify the characteristics of individuals.

Nevertheless, biological impedance has not been realized as a biometric technology for personal identification owing to its low reproducibility^31^. Compared to other basic criteria for biometric security systems such as uniqueness, universality, collectability, performance, acceptability and circumvention^32^, the major obstacle for biological impedance is permanence^31^. Every single characteristic or trait recorded in the database requires to be constant over a period. However, no matter how precise a system detects electrical impedance of fingers, the variation due to extraneous and inherent factors of normal physiology can inevitably affect the ability of identifying individuals. The impedance varies greatly depending on various factors such as skin moisture, body fat, and blood vessel expansion due to body temperature^33^. The temperature-induced change in resistance could be due to alterations in cutaneous blood flow or compartmental distribution of body water^34^. Several previous studies have revealed the relationship with the relevant factors. For instance, Deurenberg *et al*.^35^ assessed the effect of ingesting a meal, drinking normal or beef tea, exercising, and the menstrual cycle on body impedance. Lim *et al*. mentioned that different physiological conditions such as thickened skin, fluid retention, and obesity can affect the impedance measurement^33^. Carton *et al*.^34^ found that varying skin temperature by altering ambient temperature significantly changes resistance measurements and the estimation of total body water. Liang and Norris^36^ revealed the effects of increased skin blood flow and skin temperature on bioelectric impedance. Gudivaka, D. Schoeller, and Kushner^37^ found that impedance varied inversely with a change in skin temperature for the frequency range of 5 kHz to 500 kHz. These findings indicate that varying skin temperature significantly changes impedance measurements. Despite studies on the factors affecting impedance measurements, there has been a lack of a systematic approach for removing the associated effects and obtaining consistent data. Therefore, the characteristic of the biological impedance itself is not stable and permanent, and therefore, its use as robust biometrics is limited.

To overcome these limitations, we propose a new ratiometric method to extract reliable features based on impedance ratios obtained by multi-channel impedance spectroscopy. As a proof of concept, a simplified bioelectrical model was used to illustrate the ratiometric features that improve performance for conditions where undesirable factors (e.g., temperature, inherent physiology) change the impedance of underlying biological tissues. To obtain multi-channel impedance spectra, we designed a system applying modulated sinusoidal current and sensing voltage across different pairs of fingers by electrically switching the five electrode pairs mounted on the board. The measurements were carried out for a total of 10 finger pairs. Our human subject test showed that the ratiometric impedance spectra improved reproducibility as well as amplified the difference between individual subjects. As a result, we confirmed that the ratio characteristics extracted by applying this method and machine learning increased classification accuracy and lowered the error rate by a factor of 5 in comparison to using original data. Taken together, these demonstrations make multi-channel impedance spectroscopy highly useful and important for the future development of extremely secured and high-performance biometrics.

## Results and Discussion

### Impedance-based identity authentication system

**Figure 1:**
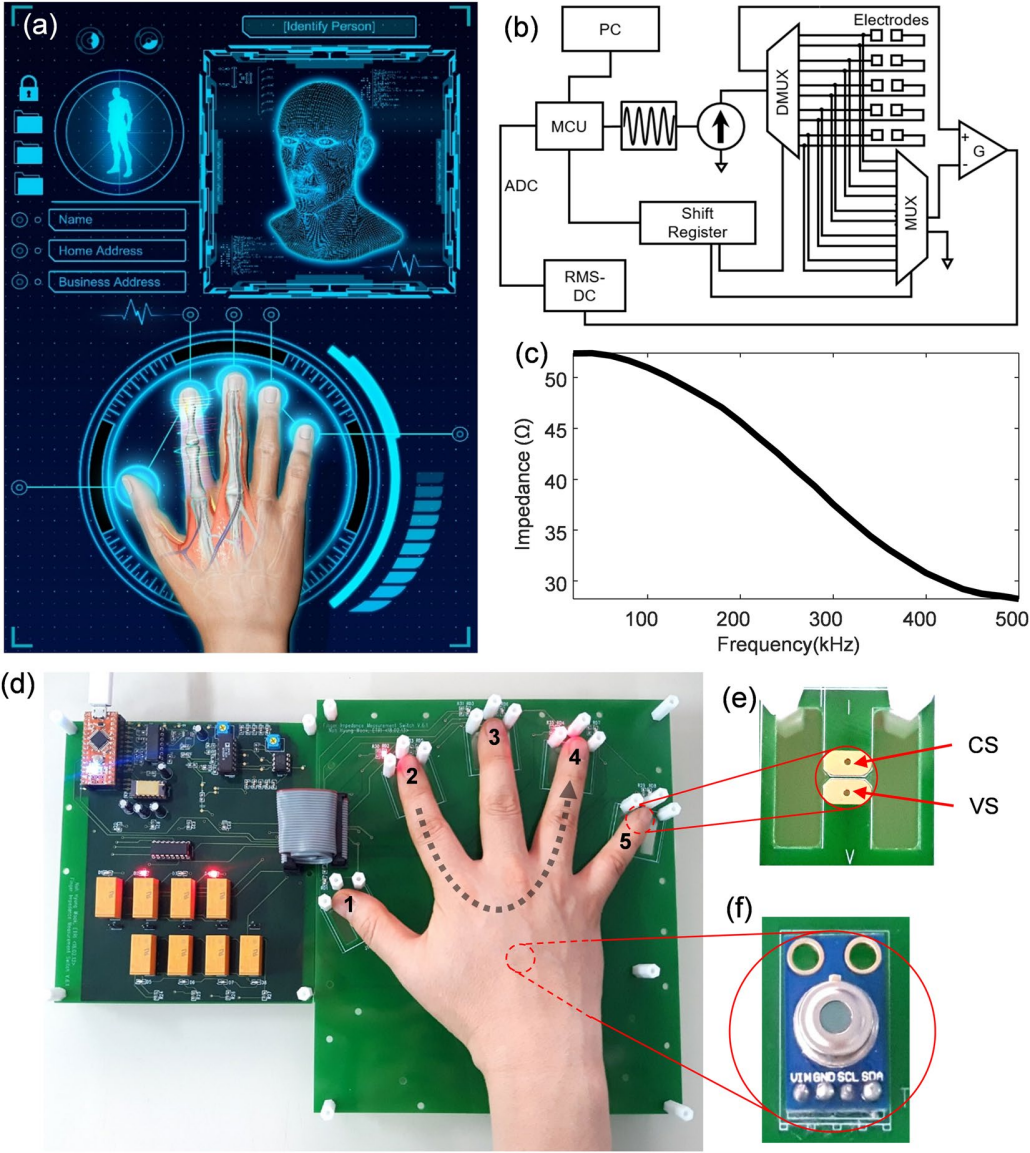
Schematic of electric impedance spectrum identity authentication system. (**a**) Concept of identity authentication system which employs the electrical transfer characteristics through finger bones and tissues. (**b**) Schematic of the finger impedance-based identity authentication system. Sinusoidal current is transmitted sequentially to the finger pairs by the operation of the multiplexer and demultiplexer, converted into a DC signal by the RMS-DC converter, and digitized by the ADC of the microcontroller. (**c**) The shape of the electrical finger impedance spectrum measured by our system. (**d**) Our developed system measuring finger impedance by placing the hand of the subject on the board with five pairs of electrodes. Eight mechanical relays mounted on the left board enable switching of current path lines in sequence to measure ten pairs of finger impedances. Numbers 1 to 5 are assigned in order from thumb to index finger. The LED lights on the right boards 2 and 4 indicate the pair of index-ring finger currently being measured. (**e**) The separate pairs of current-sourcing (CS) and voltage-sensing (VS) electrodes to apply 4-point electrode measurement. (**f**) The infrared thermometer sensor is mounted on the board to measure the temperature of the subject’s hand.

Human body tissue is composed of conducting ionic electrolytes and poorly conducting cells and extracellular matrices (e.g., skin, fatty tissues, bone, cartilages), resulting in both resistive and capacitive properties. Therefore, electrical signals transmitted through the fingers provide anatomical information about the fingers and relevant biomaterials and bioelectrical properties. To implement identity authentication based on the electrical properties of human body tissues, we designed a system that detects electrical impedance between two fingers by applying modulated sinusoidal current (Fig. 1(a)). The system comprises a microcontroller, waveform generator, and constant current source that can be switched by a multiplexer so that the measuring electrode pairs can be reconfigured. The schematic of our impedance measurement system is illustrated in Fig. 1(b). Figure 1(c) shows an electrical impedance spectrum measured by our system; the impedance decreases with increasing frequency.

For identity authentication, a user places five fingers on the multi-sensing system with five pairs of electrodes arranged as shown in Fig. 1(d). The electrodes are located at the fingertip and the constant source current is passed through the pairs of two fingers being measured. As the current flows through the pairs of fingers, the voltage across the pair is measured by a voltage sensing electrode located between electrical paths (Fig. 1(e)). Impedances are measured for a total of 10 finger pairs. The electrical impedance spectrum for each finger pair was measured in the frequency range of 20 kHz to 500 kHz. We also mounted the infrared temperature sensor on the board to obtain the temperature of the subject’s hand (Fig. 1(f)).

### Biological Impedance variation by undesirable factors

**Figure 2:**
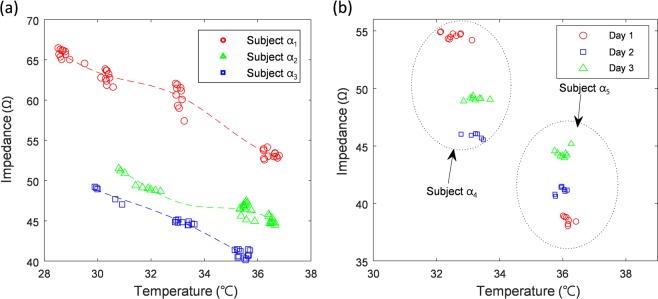
Impedance variation according to temperature. (**a**) The relationship between finger impedance and hand temperature measured in each of three subjects (α_1_~α_3_). (**b**) Impedance variation of two subjects (α_4_, α_5_) measured on different days with hand temperature controlled. The impedance in Y-axis of (**a**,**b**) is an average in the frequency range from 20 kHz to 500 kHz.

As described above for the low reproducibility of impedance measurements of the human body, the impedance of our body varies greatly depending on various undesirable factors. For those reasons, we tested the undesirable factors affecting impedance-based identity recognition. These factors can be classified into two: (i) changes in external environments (e.g., temperature, humidity) and (ii) inherent changes of normal physiology. For the external environmental factor, we examined the effect of body temperature on the impedance across fingers. An external temperature chamber (S/SM-3200, Thermotron, USA) was used to control the hand temperature between 29 °C and 37 °C for three healthy adults. After controlling the temperature of the hand using the chamber, the actual temperature of the hand was obtained simultaneously with the impedance measurement using the temperature sensor mounted on the board. In this way, we repeatedly changed the temperature of the subject’s hand and performed the impedance measurement. To analyze the change in impedance with temperature we used the average of all the impedance values obtained in the frequency range from 20 kHz to 500 kHz. As shown in Fig. 2(a), the impedance values of all three subjects changed inversely with the measured hand temperature. This result indicates that the change in body temperature is an important factor that affects electrical impedance measurement. However, as mentioned in other studies above, there are several other factors that affect biological impedance. To see the effect of these other factors, we performed day-by-day impedance measurement for two subjects at the same hand temperature over three days. Figure 2(b) shows that the temperature of the hand was constantly controlled, but the impedance values of the two subjects changed according to the date of measurement. It can be seen that the impedance can be changed by other undesirable factors, even if the temperature is controlled constantly.

### Enhanced ratiometric features reducing undesirable variation

**Figure 3:**
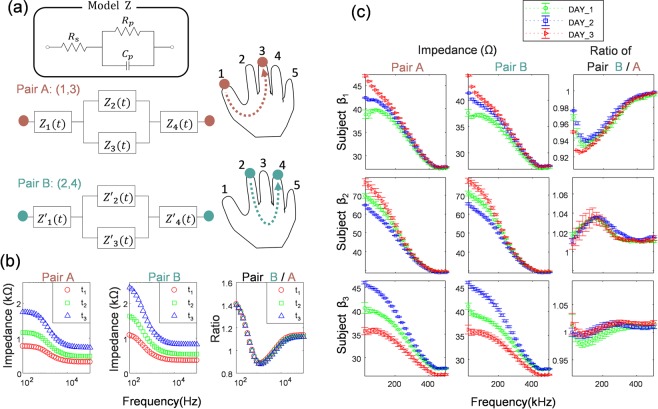
Effect of ratiometric features and bioelectrical modeling of fingers. (**a**) The bioelectrical impedance model of each finger in Pair A and Pair B designed based on the model Z. (**b**) The simulation results. The variation of each impedance curves of Pair A, B (Two left graphs) and extracted ratiometric features (rightmost graph), according to three different states (*t*_*1*_ ~ *t*_3_). (**c**) Variation of the impedance data and ratiometric features for three subjects (β_1_ ~ β_3_) measured for independent three days. The data is presented as mean ± standard deviation (n = 10).

To remove the effect of undesirable factors and obtain consistent data, we devised a method of transforming two raw impedance spectra into ratiometric traits. The rationale behind this method is explained by following the electrical model of fingers using passive components. The basic electrical model of bio-impedance is designed with the model *Z*, as shown in Fig. 3(a) ^38^. *R*_*s*_, *R*_*p*_, and *C*_*p*_ represent the electrical resistance of liquid outside the cell (in addition to the skin resistance for *Z*_1_, *Z*_4_), the resistance of intracellular fluid (ICF), and high frequency conductance of the cell membrane, respectively. When the current with low frequency less than 10 kHz is applied to the cell, the current only flows through the interstitial fluid, i.e. the ECF, but when the current with high frequency above 100 kHz is applied to the body, the current flows in both the ECF and the ICF^39–42^. The overall impedance model we used refers to the Equivalent circuit of electrode and the skin proposed for measuring bioelectric impedance^39^ consisting of electrode, epidermis, and subcutaneous layer, and we slightly modified this circuit to model a pair of fingers where epidermis is interfaced with a pair of electrodes, one corresponding to the current source and the other corresponding to the current drain. We employed a simplified model assuming 4 *Z* blocks consisting of a resistor in series with a resistor and a capacitor in parallel represent epidermis interfaced with the electrode (*Z*_1_, *Z*_4_), dermis (*Z*_2_), and sweat glands and ducts (*Z*_4_).We hypothesized that the electrical properties of all finger components would change at the same rate when the electrical properties of the hands were varied because of various environmental factors such as external temperature, internal and external moisture, and changes in blood flow. *Z* models (*Z*_1_ *~ Z*’_4_) constituting Pairs A and B were simulated to vary at a constant rate according to time *t*, and the impedance of three different states (*t*_1_ *~ t*_3_) were obtained. It can be seen that the impedance curves of Pair A and B in Fig. 3(b) change according to (*t*_1_ *~ t*_3_). The ratiometric data obtained by deriving the ratios of different impedances for each state (*t*_1_ *~ t*_3_) in Pairs A and B are shown in the rightmost graph in Fig. 3(b). It can be seen that the ratio curves of the respective state are almost coincident and overlap in the same pattern.

Figure 3(c) shows the impedance data measured for three subjects in total for 10 times per day, and the data were expressed by mean and standard deviation. We assigned numbers 1 to 5 in order from thumb to index finger. Pairs A and B in Fig. 3(c) represent finger pairs of thumb-middle and index-ring, respectively. The measured data for the three subjects also showed impedance variation by the date of measurements. As a result of transforming two raw impedance spectra showing difference by date of measurement into the ratiometric features (ratio between the impedances), it was confirmed that highly reproducible curve characteristics for each subject can be obtained.

### Improved discerning of difference between pairs of fingers by ratiometric features

**Figure 4:**
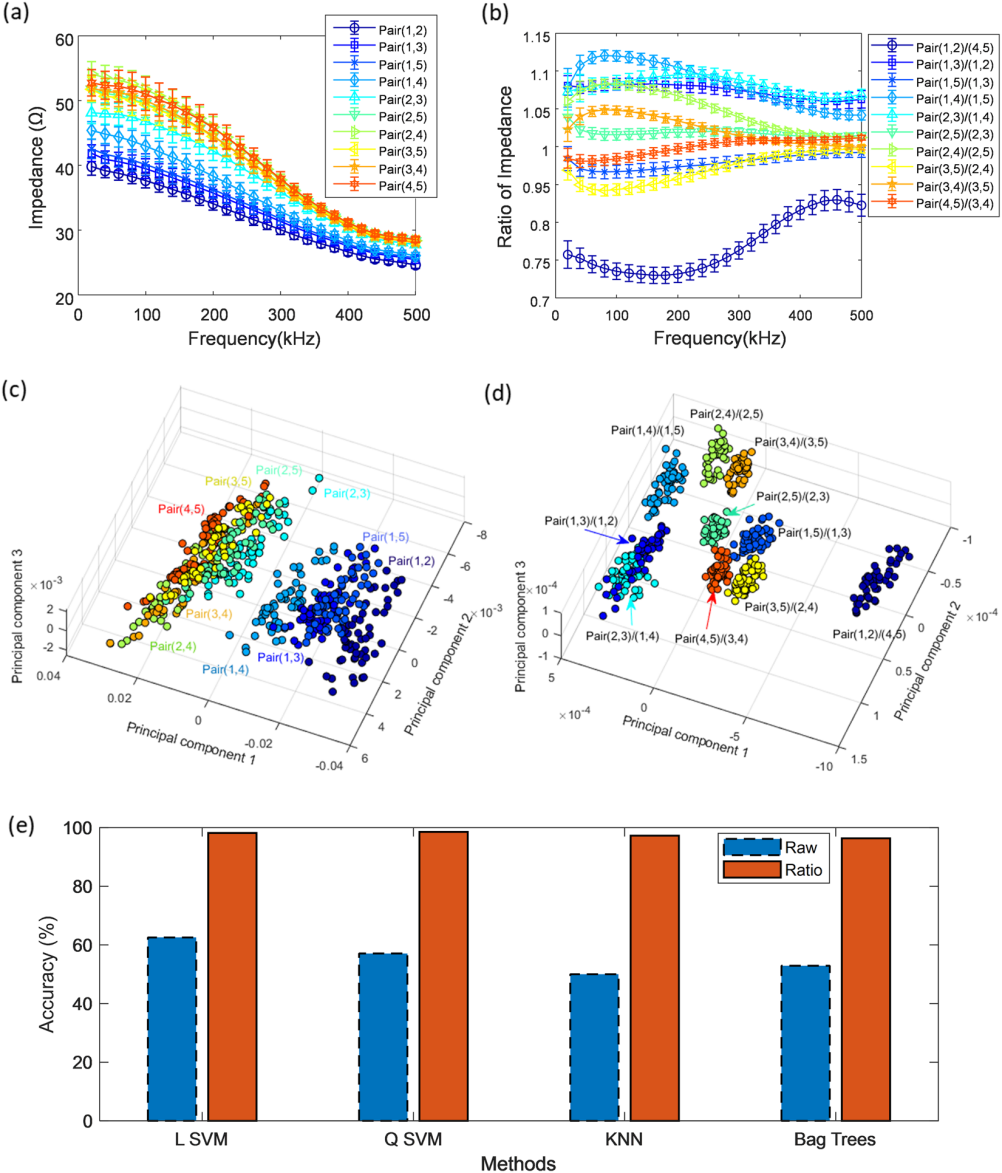
Classification of different finger pairs based on raw data and ratiometric features. (**a**) The raw data of the measured impedance of finger pairs. (**b**) The ratiometric features derived by transforming raw impedance of finger pairs. The error bars in the figure indicate the relative standard deviation. (**c**) Scatter plot of the first three principal components of the raw data of impedance from principal component analysis. (**d**) Scatter plot of the first three principal components of the ratiometric features from principal component analysis. (**e**) Comparison of classification result of raw data and ratiometric features of 10 finger pairs.

As a demonstration of the reproducibility enhancement by using ratiometric features, we tested the distinguishability of finger pairs. Since the anatomical configuration of each finger is slightly different, it can be expected that the impedance tendency will be different in each finger pair. Therefore, we analyzed each impedance data of finger pairs in various models. Ten pairs of fingers can be formed by selecting two of five fingers. Thus, we measured the impedance of ten pairs of fingers in sequence by electrically switching the five electrodes mounted on the board. We performed the impedance measurements for one subject 10 times per day and data were collected on five independent days. The raw data of the measured impedance of all finger pairs shows a tendency to decrease with frequency, as shown in Fig. 4(a), and since the magnitude and slope of each finger pair are slightly different, it is possible to distinguish the finger pairs visually.

Figure 4(b) shows the impedance ratio curve obtained by applying the ratiometric method to the measured impedance of the finger pair. The 10 combinations were chosen so that each finger pair was selected only once for the numerator and denominator, respectively. To balance the scale of features, we chose the denominators that yielded the smallest deviation from the average of ratiometric features among 10 finger pairs. The extraction of the ratiometric features is described in detail in Methods. Compared to Fig. 4(a,b) shows that the patterns and slopes of the impedance ratio curves for each pair are more varied, which makes it easier to distinguish. To visualize the distinguishability of the electrical impedance of each finger pair, principal component analysis was conducted among the ten different pairs, and the first three principal components of the spectra for the both raw data and ratiometric features are displayed in Fig. 4(c,d), respectively. It can be seen that the principal components were more clearly clustered in ratio features than the raw data.

For classification of ten finger pairs, discriminative classification models were exploited. We tested multiple machine learning algorithms of linear support vector machine (LSVM), quadratic SVM (QSVM), k-nearest neighbor (KNN), and ensemble-bagged trees (Bag Trees) (Fig. 4(e)). For robust evaluation of classification accuracy, leave-one-session-out cross-validation was conducted, so that one session data of a person on a day were separated from the original dataset and used as a validation sets and all sessions were evaluated exhaustively. Figure 4(e) shows the result of applying various machine learning methods to the both raw data and ratiometric features. The experimental results indicate that the performance of the discerning of difference between the pairs of fingers can be improved using ratiometric features.

### Interpersonal difference of ratiometric impedance features

**Figure 5:**
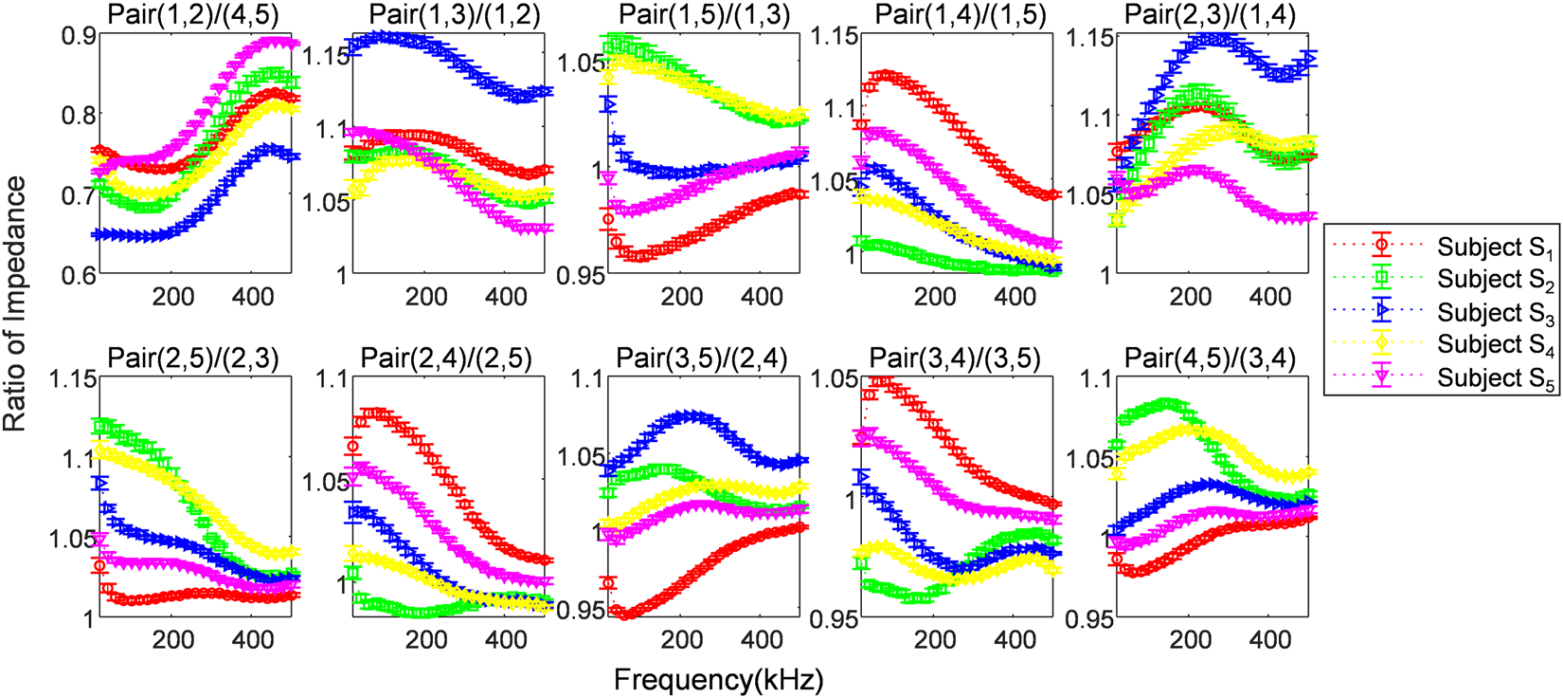
Ten pairs of ratiometric features. The ratiometric features were derived by transforming the raw impedance spectra measured in five subjects over five days. Values are the mean ± S.D. (at least n = 50).

We collected data from a group of 41 subjects to evaluate the identification performance of biological impedance spectroscopy. Subjects participated in at least five independent sessions over three months and provided at least 10 measurements per day. The raw data of the measured impedance of 10 finger pairs for five subjects are shown in Supplementary Fig. S1. In each finger pair, the raw data of all five subjects showed a gradual decrease with increasing frequency and had a similar slope. Specifically, Supplementary Fig. 1(a) shows that the raw data for the Pair (1, 5) of subjects S_1_, S_3_, and S_4_ are mostly superimposed at all frequencies, making it difficult to visually discriminate clearly. In contrast, ratiometric features have distinct patterns and slopes per finger pair (Fig. 5), and visually it is easier to distinguish than the raw data. The data for each subject also differs slightly for each finger pair in the patterns and magnitudes. It can be expected that a higher number of finger pairs improve the accuracy of discrimination. In this sense, the experiment result has the implication that the ratiometric feature is robust to the influence of environmental factors, which improve the discrimination based on reliable properties.

### Identification of individuals

**Figure 6:**
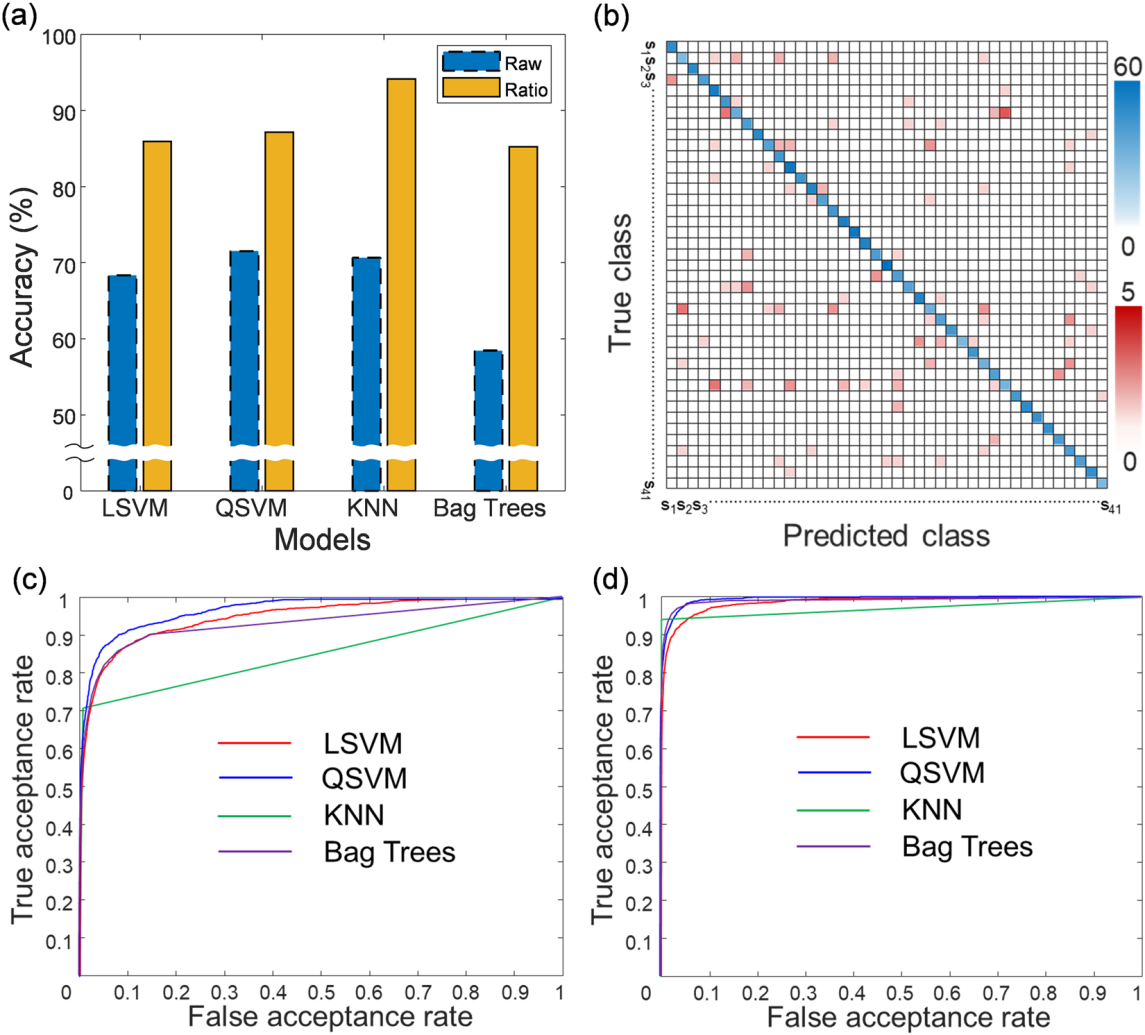
Identification accuracy of individuals by raw data and ratiometric features. (**a**) Comparison of identification accuracies for raw data and ratiometric features in the four different classification models. (**b**) Confusion matrix of the classifier of KNN among 41 subjects (S_1_ ~ S_41_), where the intensity of the color of the blue box indicates the correct predictions and that of the red boxes indicates the incorrect predictions. (**c**) ROC curves of the raw impedance data for classifiers of LSVM, QSVM, and KNN, and Bag Trees. (**d**) ROC curves of the ratiometric features for each classification model.

For the identification of individuals, we applied the discriminative classification models of linear support vector machine (LSVM), quadratic support vector machine (QSVM), K-nearest neighbor (KNN), and ensemble-bagged trees (Bag Trees) used in the previous experiment to verify the pairs of finger classification. The 10 combinations of finger pairs for ratiometric features we used in identification of subjects were the same as in Fig. 5. We used all 10 combinations of finger pairs because no particular combinations were found to show significantly distinct performance or particularly better results than other combinations. Among 41 subjects with 2166 datasets, we achieved the highest classification accuracy of 94.14% with KNN, and it was confirmed that the accuracy was improved in all tested models by applying the devised ratiometric method. The ratiometric features enhanced the accuracy of classification from 70.68% to 94.14% in the KNN model. Figure 6(a) compares the prediction accuracy for raw data and ratiometric features in the four different models. Figure 6(b) shows the confusion matrix of 41 subjects using the ratiometric features in the KNN model, where the intensity of the color of the blue box represents an accurate prediction while that of the red box represents a false prediction, and ‘S_1_, S_2,_ S_3_, ··· S_41_’ represents each subject class. The receiver operating characteristic (ROC) curves of raw data and ratiometric features for each classifier are represented in Fig. 6(c),(d), respectively. An equal error rate (EER) and an area under ROC curve (AUC) of raw and ratiometric data for the tested classifiers are listed in Table 1. The quadratic SVM classifier produced the highest AUC of 0.9939, while the KNN classifier achieved a better result in classification accuracy of 94.14% and the lowest EER of 3.0049. All EER and AUC values derived from the four models are better in ratiometric than in raw data.

**Table 1:** Comparison of raw data vs. ratiometric features.

Classifier	Raw data		Ratiometric features	
	EER (%)	AUC	EER (%)	AUC
*LSVM*	11.2896	0.9503	5.6448	0.9864
*QSVM*	9.1785	0.9701	3.8515	0.9939
*KNN*	15.0248	0.8498	3.0049	0.9737
*Bag Trees*	12.4593	0.9283	3.1981	0.9918

## Conclusion

Our research has significance as a biosignal-based authentication technology to overcome the risk of duplication of existing image-based technology including fingerprint, and to solve the existing limitations such as lack of reproducibility. The growth in use of biometric systems increases the attempts to fake the biometric systems and the biometric systems are quite susceptible to the sophisticated spoofing attacks. In recent years, biometric security against various types of attacks has been a very active field of research. This interest has led to remarkable advances, such as the development of liveness detection, for biometrics applications and the security enhancements^43^. The liveness detection techniques were used to detect spoofing attempts by determining whether a biometric object is a real human or fake. However, despite these notable progresses, the establishment of concrete protection against the threats remains a challenging task. One of the limitations of most anti-spoofing methods is the lack of generality. Although one approach may show very high performance for detecting certain types of spoofing against gummy fingers, gelatin, or playdoh copies, its performance could significantly drop when other types of synthetic features are presented^44^. In spite of a great amount of research and remarkable achievements in the field of spoof detection, the attack methodology has also evolved and become more sophisticated, so that there are still serious challenges to be faced in the detection of direct attacks^44^. On contrary, our approach is to learn the characteristics of the impedance of a finger that can classify a particular individual. These traits reflect the anatomical structure as well as biomaterial properties of the subject’s body (e.g., numerous cells and muscles, fat, ligaments and cartilage, multiple layers of tissues). Although our method does not need to learn and see fake copies, it becomes naturally able to distinguish the fake copies. For instance, in order to attempt a spoofing attack on a fingerprint system with an impedance sensing method suggested by Martinsen *et al*.^45^, it only needs to find an appropriate material that emulates electrical characteristics. However, in our case, the spoofing attempt must mimic the anatomical and biomaterial characteristics of a particular subject, which can offer a much higher level of security.

A typical liveness detection technique requires additional identification methods and thus it is only a part of the onboarding process for other biometric authentication methods. On the other hand, our method not only verifies the liveness of a subject by extracting the features of constituents inside live fingers, but also performs multiclass classification based on the characteristics of the subject. Additionally, our research suggests a solution that can overcome the limitations of low reproducibility to be used for biometrics by introducing a ratiometric feature that is robust to undesirable environmental changes and diversifies the feature patterns. Our method can be used in combination with fingerprint or other biometrics technology, yet it may also provide a unique utility with better performance than simple liveness detection.

Fingerprint is the most widely used authentication technology at present and is very unique with high accuracy of identification. For instance, the state-of-the-art fingerprint technologies (<1% EER^46^) is more accurate than our current results (~3% EER). However, the concept of this study is relatively new than modern fingerprint recognition and it is meaningful in comparison to the fingerprint recognition rate in the literature of recent decade (0.5~20% EER^46^). The empirical evaluation of uniqueness with the number of people we have demonstrated here may require further experimentation for better representation of the target population. It can also be further generalized by the theoretical evaluation using probability and statistical theory similar to DNA typing^47^ and fingerprint matching^48^. The results reported here provide future implications for biometrics as well as further broad applications of home-based healthcare environments^49^ and human-machine interface technologies such as smart watches or wearable electronics^50^.

## Methods

### Impedance measurement system

The impedance spectrum measurement system was designed to measure the impedance of ten pairs of fingers sequentially by electrically switching the five electrodes mounted on the board in the frequency range from 20 kHz to 500 kHz. Therefore, a total of 25 impedance values were obtained for each finger pair, and we used 250 feature vectors directly as inputs to the machine learning algorithms. To implement the impedance measurement system, we designed a multi-frequency constant current source. Our system included an MCU (Arduino Nano, Italy), a programmable waveform generator (AD9833, Analog Device), and a voltage-controlled current source (VCCS). The MCU was used to program the AD9833 in order to produce a multi-frequency sinusoidal signal via a serial peripheral interface. Then, this signal was converted to constant current using a VCCS-based enhanced Howland current pump^51^. We designed the system for multi-channel measurement, and eight mechanical relays were used to select two of the five electrode pairs sequentially, and a total of ten pairs of finger impedances could be obtained. These multiple relays were controlled using an 8-bit shift register (SN74HC595, TI, USA). Through this switching mechanism, our current source circuit lines were connected to two pairs of electrodes in sequence: two current-sourcing electrodes for current generation at multiple frequencies and two voltage-sensing electrodes for impedance measurement. The electrodes, which we designed by ourselves, were fabricated as double layer PCBs, and the top layer of the PCB was gold plated, and each was connected to the bottom layer through via hole. In order to make the contact area of the electrodes for each subject constant, the radius of the electrode is designed to be 0.5 cm, which is about from 50 to 70% of the area of contact with a general finger. The current source delivers a 100 µA sinusoidal current to the finger at frequencies between 20 kHz and 500 kHz. By applying a 4-point electrode measurement, ideally the impedance value is, in principle, not influenced by skin impedance. However, experimentally, there was a slight change in the impedance value depending on the skin hydration or the contact pressure in the low frequency region especially below 20 kHz. Therefore, in order to minimize these influencing variables in terms of basic research, data were acquired at frequencies above 20 kHz. Regarding the use of frequencies up to 500 kHz, the hardware we have implemented has generated stable and clear signals up to 500 kHz with neither noise nor distortion, and we determined that frequency was high enough based on the fact that several previous studies^52–54^ used the frequency up to 500 kHz to measure the constituent (e.g., intracellular fluid) associated with high frequencies for body impedance. R. Splinter^55^ also reported that 500 kHz frequency is sufficiently high for estimation of total body water including ICF in dual-frequency bioelectric impedance analysis. After the voltage electrodes sense voltage levels between two distinct positions in the current path, the measured voltage is converted to a DC signal by the RMS to DC converter (AD536AJD, Analog Devices, USA) and then collected through AD conversion. To ensure the repeatability of the measurement, the position of the fingers was fixed by using supporters at the upper part of each electrode. The infrared temperature sensor (MLX90614, Melexis, Belgium) was mounted on the board and used to acquire the temperature of the subject’s hand. The acquired impedance spectrum and temperature data were transferred from the microcontroller to a computer via USB serial communication.

### Human finger measurement of electrical impedance spectrum

The Institutional Review Board of the Ministry of Health and Welfare of the Republic of Korea approved the study and we obtained written informed consent forms from all study participants. All experiments were performed in accordance with relevant guidelines and regulations. The experiments were conducted on 41 subjects in the designated laboratory space of our institute from September through December 2018. Subjects visited only once a day, participated in at least five days over three months, and provided 8~10 measurements for each session within an hour per day. The reason for taking 8~10 measurements for each session was to obtain various training data by repeating the process of placing the subjects’ fingers at each recording. This process is similar to the fingerprints registration process, which requires repeated measurements approximately 10 times with different positions and angles. We scanned the impedance for the frequency range from 20 kHz to 500 kHz with increments of 20 kHz. For characterization of the impedance spectrum of different finger pairs, a total of ten pairs of finger impedances were obtained and principal component analysis of the impedance spectra of each finger pair was conducted by using MATLAB (R2018b, MathWorks).

### Bioelectrical modeling of human finger

The impedance of cellular tissue can be modeled as a resistor (representing the extracellular path) in series with a resistor and a capacitor in parallel (representing the intracellular path). The tissue structure and its contents may exhibit different electrical characteristics. To illustrate the electrical variation of bioelectrical impedance due to external environmental factors, the electrical finger impedance model was designed and simulated using Multisim 14.1 (National Instruments, USA).

### Ratiometric features

The term of ratiometric method is commonly used the optical and electrical sensing technologies that use the ratio between two measured signals (e.g., fluorescence intensities) that better represent the phenomenon of interest^56,57^. For instance, ratiometric method was defined previously by the use of ratios between the electrical measurements of supply voltage and output voltage levels^58^. The term “ratiometric method” in this study is defined as the use of ratios between the impedance values of two finger pairs. Specifically, the ratio is obtained by dividing the recorded value of a particular finger pair by the value of another finger pair obtained from the same measurement at the same frequency.

The measurement using our system could extract 45 possible combinations of finger pairs by selecting two out of 10 finger pairs. For the ratiometric features, 10 combinations were selected so that the combinations of finger pairs should take each finger pair only once for the numerator or the denominator, respectively. Here, the impedance values of the 10 finger pairs largely varied as shown in Fig. 4(a), and therefore the ratio values of each combination spanned very different ranges within a subject. Generally, normalizing the features is not only important if we are comparing measurements that have different scales, but it is also a general requirement for many machine learning algorithms. For machine learning, data normalization is required when features have different ranges. In addition, measured values having different scales do not contribute equally to the analysis and can cause bias^59–61^. Therefore, in order to obtain the features with a balanced scale, we chose the 10 ratiometric features so that each of the 10 finger pairs is combined with a finger pair that results in the smallest deviation from the average of other ratiometric features. To the end, the chosen ratios were pair(1,2)/pair(4,5), pair(1,3)/pair(1,2), pair(1,5)/pair(1,3), pair(1,4)/pair(1,5), pair(2,3)/pair(1,4), pair(2,5)/pair(2,3), pair(2,4)/pair(2,5), pair(3,5)/pair(2,4), pair(3,4)/pair(3,5), and pair(4,5)/pair(3,4). The ratiometric features contained a total of 250 elements, which is the same number as raw impedance data, and they were used as input to the classification algorithm.

### Recognition of individuals

For the identification of individuals, discriminative classifiers (LSVM, QSVM, KNN, and Bag Trees) were implemented by using MATLAB multiclass classification. We employed SVM with a box constraint C of 1.0 and a kernel scale of 16 for both linear and quadratic kernels. The hyper parameters were set by geometric progression from 0.5 to 32 by a factor 2. They were trained with the sequential minimal optimization algorithm^62^. kNN classifier estimates the class label of a new observation by the majority class of the k nearest neighbors. The number of nearest neighbor was set to 1 after parametric search from 1 to 20 and Euclidean distance metric was used. Bagging tree ensemble uses a collection of weak learners based on decision trees generated by a bootstrap method. We used bagging tree with Gini diversity index for split criterion, the number of trees of 30, the maximum number of decision splits of 2165, and otherwise default features. The hyper-parameters were chosen after grid parameter search among 15, 30, 45, and 60 for the number of trees and among 270, 540, 1082, and 2165 for the maximum number of decision splits. The search spaces of the hyper parameters were referred from James *et al*.^63^ and the default values in Matlab. To validate classification accuracy, each classifier was evaluated using leave-one-session-out cross-validation for 2,166 datasets from 41 subjects. Each measured spectral data was assigned to the subject class only when the data had a posterior probability greater than the threshold using the threshold score of the latent variable of the classifier. This method allows to recognize attempts by unknown users who were not registered in the training phase; thus, it may cause additional errors that predict whether the measurement belongs to more than one subject or no one. By changing the threshold value, we evaluated sensitivity (=1 - false acceptance rate) and specificity (=1 - false rejection rate) for the receiver operating characteristic (ROC) curve. The ROC curve was then used to evaluate the area under ROC curve (AUC) and an equal error rate (EER) at which the false acceptance rate (FAR) equals the false rejection rate.

## Supplementary information

Supplementary Info

## Data Availability

The datasets generated and/or analyzed during the current study are available from the corresponding author on reasonable request.
