# Supplementary material for: Ratiometric Impedance Sensing of Fingers for Robust Identity Authentication
Source: Sci Rep. 2019 Sep 19;9:13566. doi: 10.1038/s41598-019-49792-9 (PMC6753141; doi:10.1038/s41598-019-49792-9)
Supplement: Supplementary file 1 — Supplementary Info [file 41598_2019_49792_MOESM1_ESM.docx]

Supplementary Materials for

Ratiometric Impedance Sensing of Fingers for Robust Identity Authentication

Hyung Wook Noh^1,2^, Chang-Geun Ahn^1^, Hyoun-Joong Kong^2^ and Joo Yong Sim^1,*^

^1^ Medical Information Research Section, Welfare & Medical ICT Research Department,

Electronics and Telecommunications Research Institute, Daejeon, 34129, Republic of Korea

^2^ Department of Biomedical Engineering, Chungnam National University College of Medicine,

266 Munwha-ro, Jung-gu, Daejeon 35015, Republic of Korea

*corresponding author: jsim@etri.re.kr


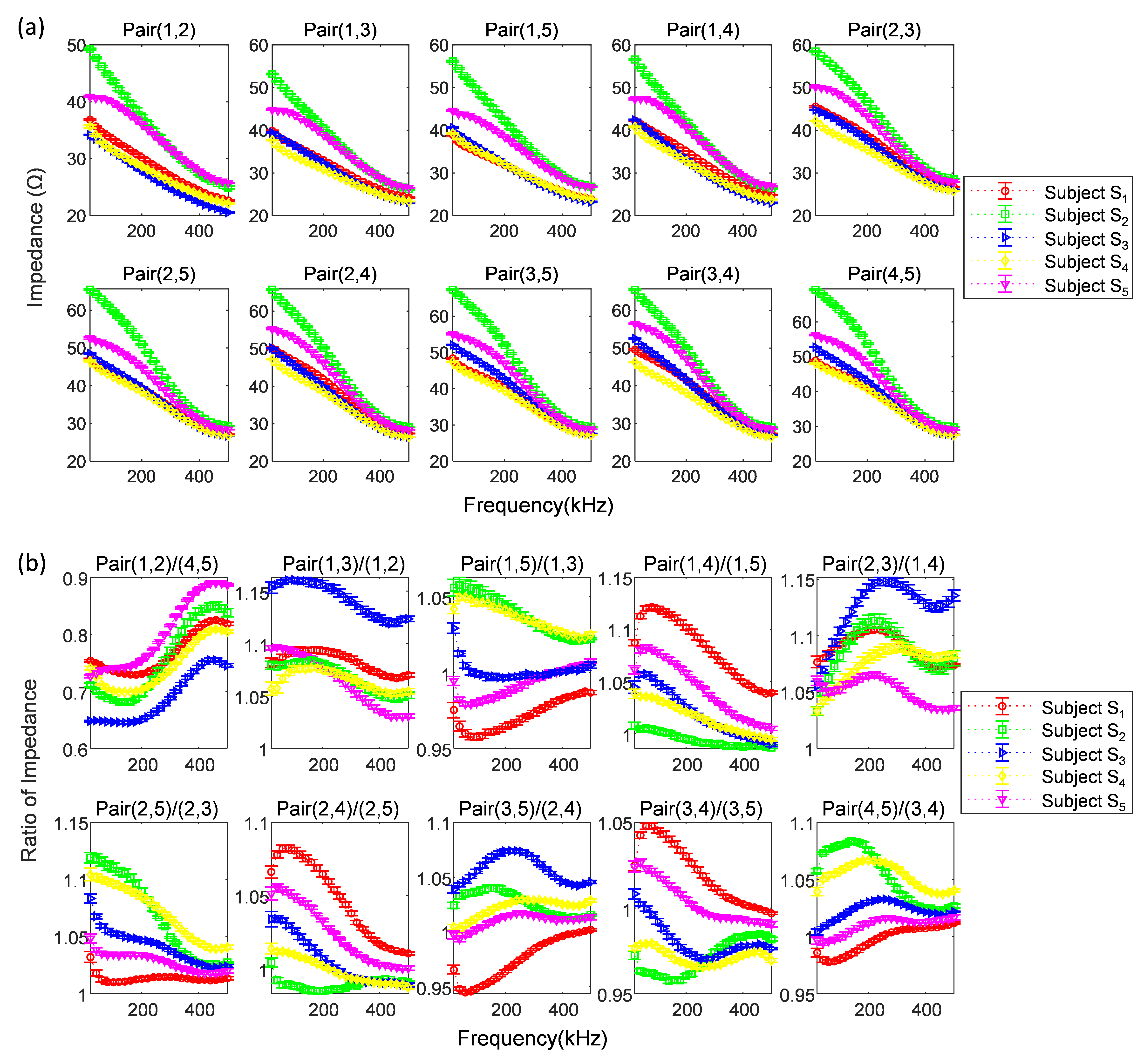


**Supplementary Figure S1. Comparison of (a) raw data and (b) ratiometric features.** The measured impedance of 10 finger pairs for five subjects over five days. Values are the mean ± S.D. (at least n = 50).
